# MEF2C Silencing Attenuates Load-Induced Left Ventricular Hypertrophy by Modulating mTOR/S6K Pathway in Mice

**DOI:** 10.1371/journal.pone.0008472

**Published:** 2009-12-29

**Authors:** Ana Helena M. Pereira, Carolina F. M. Z. Clemente, Alisson C. Cardoso, Thais H. Theizen, Silvana A. Rocco, Carla C. Judice, Maria Carolina Guido, Vinícius D. B. Pascoal, Iscia Lopes-Cendes, José Roberto M. Souza, Kleber G. Franchini

**Affiliations:** 1 Department of Internal Medicine, School of Medicine, State University of Campinas, Campinas, São Paulo, Brazil; 2 Department of Medical Genetics, School of Medicine, State University of Campinas, Campinas, São Paulo, Brazil; University of Cincinnati, United States of America

## Abstract

**Background:**

The activation of the members of the myocyte enhancer factor-2 family (MEF2A, B, C and D) of transcription factors promotes cardiac hypertrophy and failure. However, the role of its individual components in the pathogenesis of cardiac hypertrophy remains unclear.

**Methodology/Principal Findings:**

In this study, we investigated whether MEF2C plays a role in mediating the left ventricular hypertrophy by pressure overload in mice. The knockdown of myocardial MEF2C induced by specific small interfering RNA (siRNA) has been shown to attenuate hypertrophy, interstitial fibrosis and the rise of ANP levels in aortic banded mice. We detected that the depletion of MEF2C also results in lowered levels of both PGC-1α and mitochondrial DNA in the overloaded left ventricle, associated with enhanced AMP:ATP ratio. Additionally, MEF2C depletion was accompanied by defective activation of S6K in response to pressure overload. Treatment with the amino acid leucine stimulated S6K and suppressed the attenuation of left ventricular hypertrophy and fibrosis in the aforementioned aortic banded mice.

**Conclusion/Significance:**

These findings represent new evidences that MEF2C depletion attenuates the hypertrophic responses to mechanical stress and highlight the potential of MEF2C to be a target for new therapies to cardiac hypertrophy and failure.

## Introduction

Hypertrophy is a common feature of many forms of heart disease. While initially an adaptive response to increased workload and injury, in the long term cardiac hypertrophy predisposes to heart failure[1], [2], [3]. At the cellular level, myocardial hypertrophy is characterized by distinct accumulation of myofibrillar proteins and organelles in cardiomyocytes, while the development of heart failure is accompanied by degeneration and loss of hypertrophic cardiomyocytes as well as interstitial fibrosis[4]. In a current view, the hypertrophy and degeneration of cardiomyocytes represent a continuum governed by patterns of beneficial and adverse signaling triggered by stimuli such as mechanical stress and neurohumoral factors[5], [6], [7].

Among the intracellular pathways that integrate mechanical and hormonal signals, MEF2 (*myocyte enhancer factors-2,* members A to D) transcription factors play prominent roles in the regulation of cardiac hypertrophy and remodeling[8], [9], [10]. In this context, many studies have shown that overall MEF2 DNA-binding activity is enhanced in cardiomyocytes in response to biomechanical and neurohormonal stimuli[11], [12], [13]. Overexpression of MEF2A or MEF2C in cultured cardiomyocytes induces sarcomere degeneration and cardiomyocytes elongation, suggesting that activation of these members may compose signaling pathways responsible for pathologic hypertrophy [8]. Accordingly, forced expressions of MEF2A, C and D in mice heart were demonstrated to be sufficient to drive intolerance to pressure overload, ventricular chamber dilation and contractile dysfunction[8], [9], [10]. There is also evidence associating MEF2 transcription factors with common forms of human heart failure[14]. Furthermore, the transgenic expression of negative dominants of MEF2 was shown to prevent chamber dilation and mechanical dysfunction, with minor effects on cardiac growth in calcineurin-induced hypertrophy[9]. Recent studies performed in mice with dominant-negative MEF2D suggested that this factor is an important mediator of the pathologic left ventricular hypertrophy, as these mice displayed no cardiac hypertrophy, fibrosis or fetal gene activation in response to pressure overload[10]. Altogether, these evidences support the idea that MEF2 factors mediate the effects of detrimental signaling pathways in response to hypertrophic stimuli. Nevertheless, the role of each specific MEF2 member, such as MEF2A or MEF2C, in cardiac hypertrophy remains unclear mainly because of the lethal cardiac phenotypes resulting from genetic deletions of these members[15], [16], [17].

To define the potential function of MEF2C in the cardiac responses to pressure overload we conceived a strategy to deplete this factor in mouse heart by *in vivo* delivery of small interfering (si)RNA. Left ventricular hypertrophy induced by aortic banding in mice was used as a model system in this study.

## Results

### Optimization of Myocardial MEF2C Silencing by siRNA

**Figure 1:**
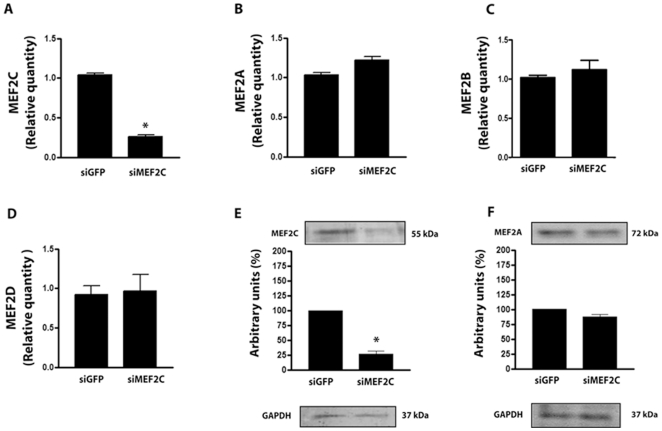
MEF2C silencing in NRVMs culture. **(A)** Bar graph shows relative quantity (RQ) of MEF2C in respect to GAPDH transcripts as a percentage of calibrator sample (siGFP) obtained by real time-PCR. **(B)** Bar graph shows the RQ of MEF2A transcripts. **(C)** Bar graph shows the RQ of MEF2B transcripts. **(D)** Bar graph shows the RQ of MEF2D transcripts. (E) MEF2C protein expression (n = 3 cultures). **(F)**, MEF2A protein expression. * p<0.05 vs treatment with siGFP. These data are from 3 NRVMs culture at least.

As an initial approach, experiments were set to test the efficacy of siMEF2C to knockdown MEF2C in cultured NRVMs (Neonatal Rat Ventricular Myocytes). Transfection efficiency of siRNA in this model system was previously assessed with fluorescent oligonucleotide[18] and was demonstrated to range around 80% in respect to cells treated with the irrelevant siGFP (Green Fluorescent Protein). Transfection of siMEF2C (300 ng/ml) reduced the MEF2C transcripts in NRVMs by approximately 80% (Figure 1A). siMEF2C had no influence in the amounts of MEF2A, MEF2B or MEF2D transcripts (Figure 1B-D). Quantitative PCRs were normalized by GAPDH (Glyceraldehyde 3-phosphate dehydrogenase). An antibody that was specific to MEF2C recognized a protein with a molecular weight close to 55KDa while specific antibody to MEF2A recognized a protein at about 72KDa in the extracts of NRVMs. Western blot analysis indicated that MEF2C was reduced in the order of 75%, while no change could be observed in the expression of MEF2A in cells treated with siMEF2C, in comparison with cells treated with siGFP (Figure 1E, F). Immunoblottings with anti-GAPDH antibody were used as control.

**Figure 2:**
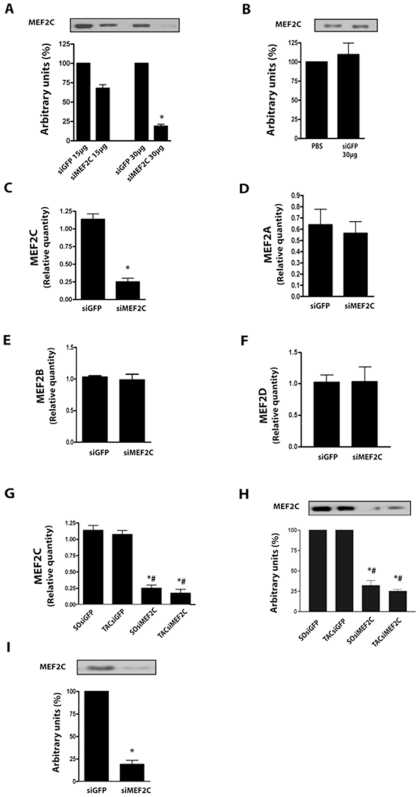
Optimization of MEF2C silencing in mice left ventricle. **(A)** MEF2C protein expression from assays with increasing amounts of siMEF2C or siGFP, via the jugular vein. **(B)** Myocardial expression of MEF2C from mice treated with siGFP and phosphate buffer saline (PBS). **(C)** Relative quantity (RQ) of MEF2C, **(D)** MEF2A and **(E)** MEF2B transcripts in mice treated with 30 µg of siMEF2C or siGFP. **(F)** MEF2D transcripts in mice treated with siMEF2C or siGFP. **(G)** Bar graphs show RQ of MEF2C transcript and **(H)** MEF2C protein expression in the left ventricle of aortic banded (TAC) and sham-operated (SO) mice. **(I)** MEF2C protein expression in adult cardiomyocytes harvested from mice left ventricle 24 hours after siRNA treatment. * p<0.05 vs SOsiGFP; # p<0.05 vs TACsiGFP. At least 6 animals were analyzed in these experiments.

After the initial characterization in NRVMs, we then searched for an amount of siMEF2C that, when injected systemically via the jugular vein, could deplete myocardial MEF2C in mice left ventricle. We started off with 450 µg/Kg of siMEF2C based on a previous report[19] and observed an expressive reduction, of about 85%, of myocardial MEF2C protein expression 1 day after bolus injections of 900 µg/Kg of siMEF2C (Figure 2A). Injections of siGFP (900 µg/Kg) did not affect MEF2C expression levels in the left ventricle in comparison with phosphate buffer saline (Figure 2B). The treatment with siMEF2C was accompanied by marked reduction of MEF2C transcripts, but not of those from MEF2A, MEF2B or MEF2D (Figure 2C-F), after normalization to GAPDH. Reduction in MEF2C transcripts and protein levels was also demonstrated to occur in banded mice treated with siMEF2C (Figure 2F, G).

Complementary data on the ability of siMEF2C to deplete MEF2C protein levels was obtained by analyzing isolated cardiomyocytes. The expression of MEF2C was reduced in cardiomyocytes harvested from the left ventricle 24 hours after the treatment with siMEF2C to similar levels of myocardial extracts (∼75%), as depicted in Figure 2H.

**Figure 3:**
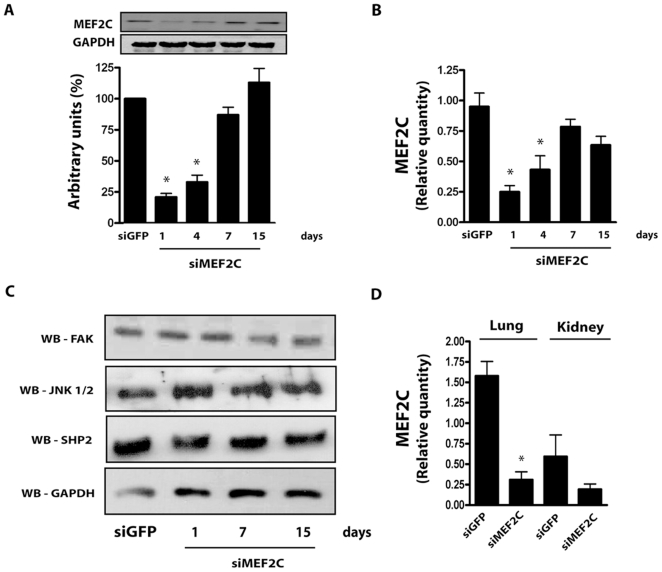
Effects of siMEF2C in distinct tissues and signaling proteins. **(A)** Time-course of MEF2C protein expression and **(B)** transcript levels normalized by GAPDH. **(C)** Myocardial expression of FAK, JNK, SHP2 and GAPDH. **(D)** Relative quantity of MEF2C transcripts in mice lung and kidney. * p<0.05 vs treatment with siGFP. At least 5 mice were employed for each subgroup.

We then set to address the time-course of MEF2C reduction in the left ventricle obtained after systemically delivered siMEF2C. As shown in Figure 3A, MEF2C protein expression was reduced by approximately 75% in the left ventricle of control mice up to the 4^th^ day after the administration of siMEF2C. However, by the 7^th^ day after the siMEF2C myocardial MEF2C reached levels comparable to those of mice treated with siGFP and remained unchanged thereafter. Further analysis confirmed similar reductions in MEF2C transcripts in the myocardium over time after the administration of siMEF2C (Figure 3B). Signaling molecules potentially involved in MEF2C activation[12], [13], [18] were also examined to exclude off-target effects of siMEF2C. As shown in Figure 3C, no change was detected in the myocardial levels of FAK (Focal Adhesion Kinase), JNK (c-Jun N-Terminal Kinase) or Shp2 (Src homology region 2, phosphatase 2) after treatment with siMEF2C.

The consequences of the treatment with siMEF2C in tissues such as lung and kidney were analyzed. We found reductions of MEF2C transcripts in the lung similar as to those of left ventricle from mice treated with siMEF2C (Figure 3D). However, treatment with siMEF2C did not change the amount of MEF2C transcripts in the kidney. Overall, the results establish that siMEF2 administered via jugular vein can silence left ventricle MEF2C protein levels, although it also depletes MEF2C in the lungs.

### MEF2C Silencing Attenuates Load-Induced Hypertrophy in Aortic Banded Mice

**Figure 4:**
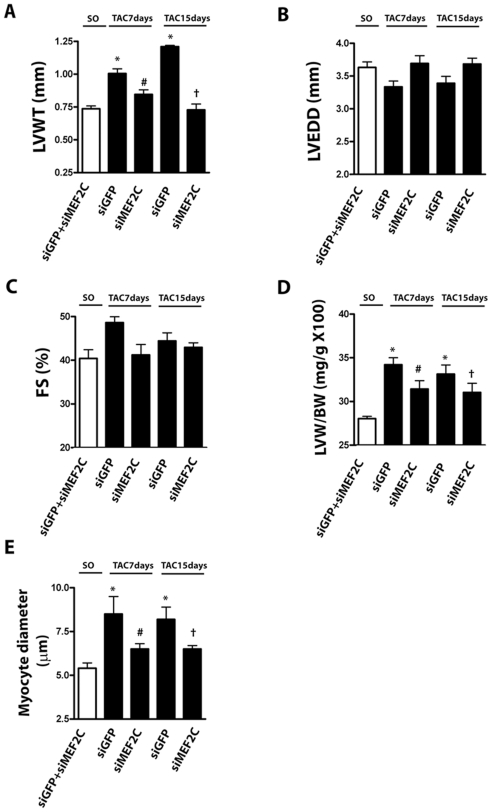
MEF2C silencing prevents load-induced left ventricular hypertrophy. Data from sham operated (SO) and aortic banded mice (7 to 15 days after transverse aortic constriction – TAC). Bar graphs indicating the echocardiographic values of **(A)** posterior wall thickness (LVWT), **(B)** diastolic diameter (LVEDD) and **(C)** fractional shortening (FS). The values of SO mice treated with siMEF2C or siGFP did not reach statistical significance, so they were averaged. **(D)** Bar graphs show the left ventricle mass/body mass ratio and **(E)** the average cardiomyocytes diameter. * p<0.05 vs SO; # p<0.05 vs TAC 7days siGFP; † p<0,05 vs TAC 15 days siGFP. A total of 9 mice were analyzed in each subgroup.

Next we investigated whether MEF2C silencing affects the load-induced left ventricular hypertrophy. In this set of experiments siMEF2C was intravenously injected in mice via jugular vein one day before sham-operation or aortic banding. We found that blood pressure, heart rate, left ventricular structure and function of sham-operated mice were unaffected by siMEF2C, indicating that depletion of MEF2C does not influence basal cardiac function or structure. Therefore, for the analysis of left ventricle echocardiography and gravimetry we considered sham-operated mice treated with siGFP or siMEF2C as a single control group. Treatment with siGFP did not affect the typical changes of the left ventricle induced by pressure overload (i.e. increases of left ventricle thickness, cardiomyocyte diameter and interstitial fibrosis). Pretreatment with siMEF2C retarded the load-induced left ventricular hypertrophy as assessed by echocardiographic determination of left ventricular wall thickness (Figure 4A). Depletion of MEF2C did not change the left ventricular diameter or fractional shortening (Figure 4B, C). Notably, the systolic gradient across the aortic constriction was similar in mice injected with siGFP or siMEF2C (Table 1). The attenuation of load-induced left ventricular hypertrophy in mice treated with siMEF2C was confirmed by gravimetry (Figure 4D). In addition, siMEF2C attenuated the increases in the diameter of cardiomyocytes (Figure 4E).

**Table 1:** Mice hemodynamics.

	Vehicle						Leucine			
	siRNA^GFP^			siRNA^MEF2C^			siRNA^GFP^		siRNA^MEF2C^	
	SO	TAC 7days	TAC 15 days	SO	TAC 7 days	TAC 15 days	SO	TAC 7days	SO	TAC 7days
**N**	11	12	7	11	7	10	9	8	7	9
**SBPa(mmHg)**	123±3	171±3*	177±4*	121±3	171±3*	171±6*	138±3	171±6#	145±4	160±6#
**SBPf (mmHg)**		119±2	128±4		124±3	125±5		123±2		129±4
**SGr**		52±2	49±3		46±1	46±5		48±5		43±2
**HR (beats/min)**	397±22	420±16	377±26	436±15	365±19	365±26	415±15	422±12	443±11	402±9

SO. Sham operated; TAC. transverse aorta constricted; SBPa. systolic blood pressure in ascending aorta; SBPf. systolic blood pressure in femoral artery; SGr. systolic gradient; HR. heart rate.

*P<0.05 indicated statistical significance compared to values of SO mice treated whit vehicle.

# *P*<0.05 indicated statistical significance compared to values of SO mice treated whith leucine.

**Figure 5:**
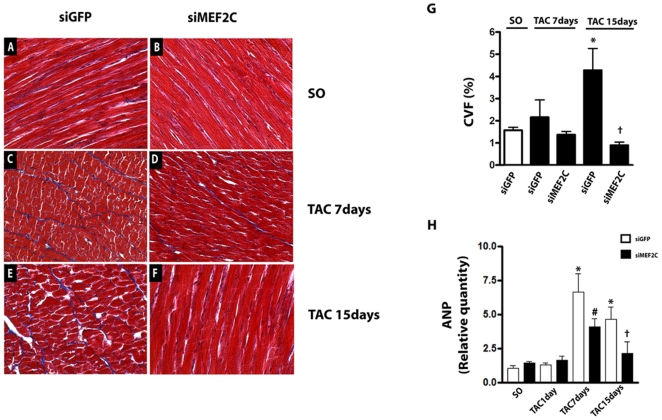
MEF2C silencing prevents the myocardial deleterious effects of pressure overload. Myocardial samples of SO and TAC mice treated with siMEF2C or siGFP stained with Masson trichrome (X400): **(A)** - SO- siGFP, **(B)** – SO- siMEF2C, **(C)** – TAC(7days)- SO- siGFP, **(D)** - TAC(7days)- siMEF2C, **(E)** - TAC(15days)- SO- siGFP, **(F)** - TAC(15days)- siMEF2C. **(G)** Bar graph indicates the average fraction volume of collagen (CVF%) (n = 9 mice each subgroup). **(H)** Relative quantity (RQ) of ANP transcript (n = 9 mice each subgroup). * p<0.05 compared with values of SO mice; # p<0.05 compared with values of TAC 7days siGFP mice, † p<0,05 compared to values of TAC 15days siGFP mice.

Representative examples of myocardial samples from sham-operated and banded mice treated with siGFP or siMEF2C are shown in Figure 5A–F. Left ventricular fibrosis was attenuated in aortic-banded mice treated with siMEF2C (Figure 5G). Figure 5H displays data of ANP (Atrial Natriuretic Peptide) transcript in the left ventricle from sham-operated and aortic banded mice treated with siGFP or siMEF2C. MEF2C silencing markedly attenuated the rises of ANP transcripts in the left ventricle of banded mice.

### MEF2C Depletion Is Associated with Deficiency in Load-Induced mtDNA Content

**Figure 6:**
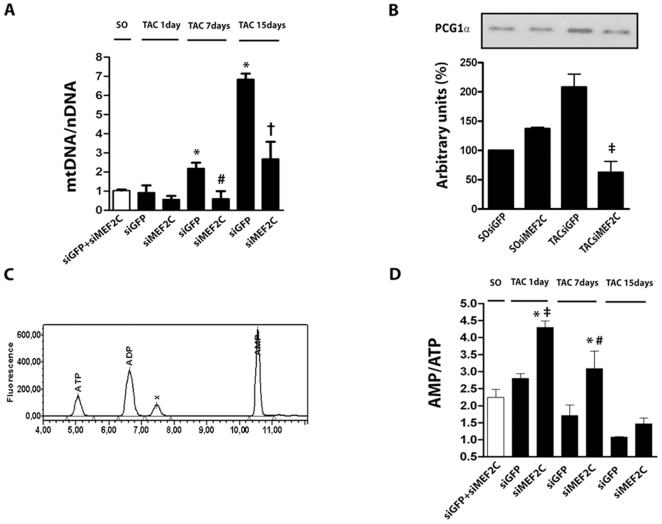
MEF2C silencing negatively regulates myocardial mitochondriogenesis. **(A)** Bar graph shows relative quantity (RQ) of mtDNA (mt - mitochondrial) to nDNA (n – nuclear). **(B)** PGC-1α protein expression. **(C)** Representative chromatogram of AMP, ADP and ATP detection peaks. **(D)** Bar graph indicates the average values of the AMP:ATP ratio. The data were from left ventricle of SO and TAC (1 day) mice treated with siMEF2C or siGFP (n = 9 mice each subgroup).* p<0.05 compared with values of SO mice; ‡ p<0.05 compared with values of TAC 1day siGFP mice; # p<0.05 compared with values of TAC 7days siGFP mice; † p<0.05 compared with values of TAC 15days siGFP mice.

MEF2 factors are important regulators of gene transcription involved in cardiac mitochondriogenesis and energy metabolism[20]. Mitochondrial proliferation is frequently accompanied by a high rate of mtDNA (mitochondrial DNA) replication[21]. Conceivably, depletion of MEF2C results in changes in the abundance of mtDNA and energy metabolism in overloaded left ventricle. Therefore, we next examined if depletion of MEF2C influences the rises of mtDNA copy number induced by pressure overload in mice left ventricle. The levels of mtDNA relative to nDNA (mtDNA:nDNA) were similar in the left ventricle of sham-operated mice treated with siMEF2C or siGFP. Thus, the data of these two groups were averaged for the comparative analysis with data from aortic banded mice. As shown in Figure 6A, pressure overload lasting for 7 and 15 days markedly increased the left ventricular mtDNA:nDNA ratio. Depletion of MEF2C markedly attenuated the rises of the mtDNA:nDNA ratio induced by aortic banding.

The augmentation of mtDNA abundance during the myocardial hypertrophic growth involves regulation of mtDNA replication by mtTFA (mitochondrial transcription factor A) by nuclear-encoded transcription factors (NRFs) and PGC-1 (peroxisome proliferator-activated receptor-gamma coactivator-1) transcriptional co-activators[22], [23]. Therefore, we next examined if depletion of MEF2C could change the expression of PGC-1α in mice left ventricle. As shown in Figure 6B, aortic constriction (1 day) induced a rapid increase in the expression of PGC-1α, an effect that was markedly attenuated by MEF2C depletion. Basal myocardial expression of PGC-1α remained unchanged in sham-operated mice depleted of MEF2C.

Given that mitochondria are the major source of energy in the myocardium, we next examined whether changes in the levels of mtDNA:nDNA were accompanied by changes in the myocardial AMP:ATP ratio, as a mean to assess the energy levels of the myocardium. Figure 6C shows a representative chromatogram used for the detection of ATP, ADP and AMP levels of left ventricle. MEF2C silencing did not change AMP:ATP ratio in the left ventricle of sham operated mice, therefore data from sham-operated mice treated with siGFP and siMEF2C were averaged. As shown in Figure 6D, the values of myocardial AMP:ATP ratio were found to be greater in the left ventricle of 1 and 7 day banded mice treated with siMEF2C in comparison to those treated with siGFP (Figure 6D). Overall, these data support the notion that depletion of MEF2C attenuates the load-induced replication of mtDNA and likely in mitochondrial biogenesis in mice left ventricle, associated to a defect in the regulation of PGC-1α expression.

### MEF2C Depletion Attenuates Hypertrophy by Modulating mTOR/S6K Pathway

**Figure 7:**
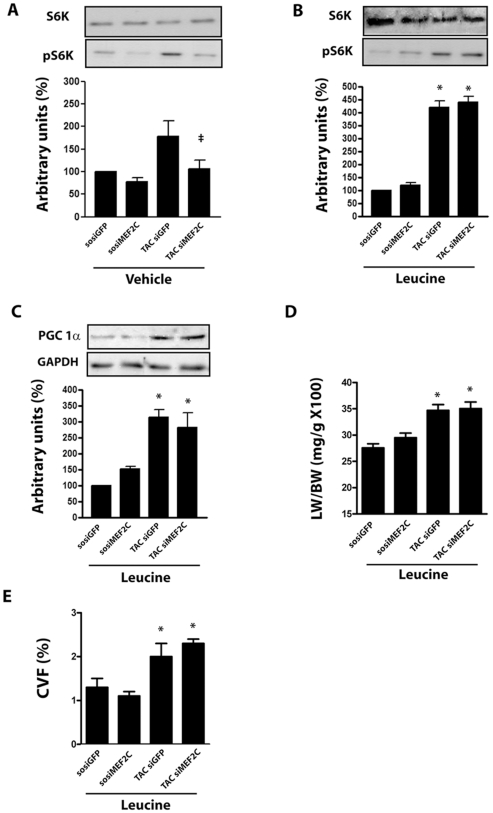
MEF2C depletion attenuates hypertrophy by modulating mTOR/S6K pathway. **(A)** S6K and phosphoS6K (p-S6K) protein expression in the left ventricle of SO and TAC (1 day) mice treated with siMEF2C or siGFP (n = 6 mice each subgroup). From samples of left ventricle of SO and TAC (7 days) mice treated with siMEF2C or siGFP and leucine (n = 9 mice each subgroup) were measured: **(B)** S6K, phosphoS6K (p-S6K) and **(C)** PGC-1α protein levels, **(D)** the left ventricle mass/body mass ratio mice and **(E)** the average fraction volume of collagen (CVF%). * p<0.05 compared with values of SO mice.

Previous studies have indicated that perturbing mitochondrial function can influence the activity of mTOR/S6 kinase, a signaling pathway critically involved in the reactive cardiac hypertrophy[24], [25]. Therefore we next examined whether the anti-hypertrophic effect of MEF2C depletion is related to a defective activation of mTOR (mammalian Target of Rapamycin). Data shown in Figure 7A indicate that aortic banding markedly increased S6K Thr389 phosphorylation in mice left ventricle, while depletion of MEF2C abolished this effect. The amino acid leucine is a potent activator of mTOR complex by a pathway distinct from TSC-2 (Tuberous Sclerosis Protein-2) complex phosphorylation[26], [27]. Thus, we reasoned that supplementation with leucine might rescue the defective activation of mTOR/S6K complex after depletion of MEF2C. Supplementation of leucine in the drinking water enhanced basal phosphorylation of S6K in the left ventricle of sham-operated mice treated with siMEF2C or siGFP (Fig 7B). Moreover, it partially restored the phosphorylation of S6K in the left ventricle of banded mice depleted of MEF2C, in parallel with the restoration of the load-induced expression of PGC-1α (Fig 7C). Finally, we found that supplementation with leucine attenuated the anti-hypertrophic effect of MEF2C depletion, as indicated by gravimetry and histological data shown in Figure 7D, E. These data are consistent with the hypothesis that a defect in the activation of mTOR/S6K pathway provides a mechanism by which MEF2C depletion attenuates the load-induced left ventricular hypertrophy.

## Discussion

In this study, we applied RNA interference technology to define the function of MEF2C in the adult heart. Our results demonstrate that the depletion of MEF2C by siRNA attenuates the hypertrophic growth of mice left ventricle in response to pressure overload, reduces hypertrophy in cardiomyocytes and diminishes interstitial fibrosis and attenuates the upregulation of ANP. These effects indicate that the downregulation of pathways controlled by MEF2C mitigates the adverse effects of pathologic cardiac hypertrophy and remodeling. Additionally, the results of the current study reveal that the anti-hypertrophic effects of MEF2C depletion may be related to a defective mobilization of mTOR/S6K by pressure overload, which may be associated with an intricate mechanism that involves negative modulation of the mtDNA replication and changes in myocardial energy metabolism caused by defective activation of PGC-1α.

### Myocardial MEF2C Depletion by siRNA

We observed a reduction of about 80% in the levels of myocardial MEF2C through siMEF2C delivery via the jugular vein. The gene silencing induced by siMEF2C was restricted to the lung and heart, indicating the efficiency of this route to achieve delivery of siRNA to the left ventricle, as well as the lack of significant exposure of tissues and organs distal to the left ventricle to siRNA. Besides the left ventricle, depletion of MEF2C was confirmed to occur in cardiomyocytes harvested from mice treated with siMEF2C, thereby demonstrating the susceptibility of this cell type to the effects of systemically delivered siRNA. Moreover, the specificity of MEF2C silencing was substantiated by the lack of effects of siMEF2C on MEF2A, MEF2B or MEF2D, as well as on the unrelated proteins FAK, JNK, SHP2 and GAPDH. The MEF2C depletion induced by the RNAi strategy used in the present study lasted for approximately 4 days. Such a prolonged effect of systemically delivered siRNA was previously observed for distinct targets in various tissues[19], [28], although the underlying mechanism remains unclear.

### Effects of MEF2C Depletion in Overloaded Left Ventricle

A key finding in the current study is the attenuation of the load-induced left ventricular hypertrophy induced by MEF2C silencing. Remarkably, our results also show that depletion of MEF2C attenuated the interstitial fibrosis and the rise of ANP expression, without affecting the left ventricular function, indicating that depletion of MEF2C may reduce the adverse effects of pressure overload. Consistent with this hypothesis, data from previous studies indicate that activation of MEF2C may mediate myocardial adverse events invoked by pathological stress[8].

MEF2 transcription factors have been implicated in the direct and indirect regulation of multiple genes[8], [9]. It has been shown that a null mutation of MEF2C affects the expression of cardiac structural and stress response genes in developing hearts[15]. In addition, studies that analyzed global gene expression in the left ventricle of MEF2C transgenic mice indicated an upregulation of genes encoding ion-handling and extracellular matrix-associated proteins[8], which might be involved in the deleterious effects of MEF2C activation. However, it is still uncertain whether these genes are involved in the hypertrophic growth induced by MEF2C activation. The data gathered in the present study reveal that MEF2C depletion blunted the increases in mtDNA and the ability of the myocardium to sustain the ATP levels when subjected to pressure overload, indicating that MEF2C may play a role in regulating the cardiac mitochondriogenesis and energetic metabolism in response to hypertrophic stimuli. Accordingly, we have shown here that MEF2C depletion markedly attenuated the rise of PGC-1α induced by pressure overload, providing an illustration of the specific contribution of the MEF2C to the control of myocardial mitochondria and metabolism in response to mechanical stress. Consistent with this, the level of PGC-1α was previously shown to play a major role in setting the cardiac mitochondrial content[22], [23]. Furthermore, MEF2 factors have been shown to mediate the Ca^2+^/Calcineurin activation of PGC-1α promoter and deletion of MEF2A in mice was demonstrated to result in perturbation of mitochondrial structure[17], [29]. Finally, forced expressions of either MEF2C or PGC-1α are known to lead to mitochondrial ultrastructural abnormalities and development of cardiomyopathy[8], [22], [23]. Taken together, these data imply that modulation of load-induced mitochondrial biogenesis may contribute to the beneficial effects of MEF2C depletion in overloaded myocardium. It was somewhat surprising to find that the improved cardiac phenotype after MEF2C, i.e. with less fibrosis and less pathological hypertrophy, was accompanied by a transient worse energetic profile including higher AMP/ATP levels. Although we have no clear explanation for these effects, one could speculate they are consistent with an expected increase in the myocardial energy consumption induced by pressure overload in the absence of appropriate hypertrophic growth. Maintenance of normal cardiac function in the face of increased workload but without changes in the left ventricle wall thickness, end-diastolic or end-systolic volumes implies a positive inotropic effect, which is paralleled by increased energy consumption per unit of myocardial mass. This would explain the higher AMP/ATP ratio of overloaded mice left ventricle silenced for MEF2C. Moreover, these data may imply that the activation of mitochondrial biogenesis in the early hypertrophic responses to pressure overload might have detrimental influence on the heart. Accordingly, increased mitochondrial mass could induce abnormally high myocardial oxidative stress that in turn might induce cell loss in the early period of pressure overload[30]. Alternatively, functional and structural abnormalities of mitochondria from overloaded heart might be related solely to increases in mitochondrial mass which might affect myofibrils, compromising the contractile function[31]. Thus it is conceivable that lessening MEF2C levels by modulating the excessive mitochondrial biogenesis of overloaded myocardium, may contribute to mitigate the degeneration of overloaded myocardium. However, this issue needs further studies.

The data from the present study indicate that MEF2C depletion is accompanied by downregulation of the mTOR/S6K pathway [32], [33] [34]. Remarkably, the restoration of the activity of this signaling complex after supplementation with leucine was accompanied by suppression of the anti-hypertrophic of MEF2C depletion, indicating that the defective activation of mTOR/S6K pathway is a critical component of the beneficial effects of MEF2C depletion in the cardiac phenotype of TAC mice. Conversely, the activation of this pathway might be involved, together with changes in mitochondrial biogenesis and function, to the detrimental effects of MEF2C activation in cardiac hypertrophy. These assumptions are in agreement with previous data indicating that mTOR/S6K complex plays a crucial role in the hypertrophic growth of cardiomyocytes and left ventricle invoked by mechanical stress[18], [35]. Moreover, mTOR overactivation has been shown to cause increased mitochondrial biogenesis and accumulation of reactive oxygen species in distinct model systems[36], suggesting that the activation of mTOR pathway might be responsible for the detrimental effects of MEF2C activation and vice-versa to the beneficial effects of MEF2C depletion in the mechanically overloaded hearts. However, the mechanisms that connect the MEF2C to mTOR/S6K pathway were not explored in the present study. It is possible that the activation of AMPK induced by the raise in the AMP:ATP relative amount may inhibit the mTOR/S6K complex in the overloaded myocardium[32]. Alternatively, reduced mitochondrial function may also inactivate mTOR activity[37], however further studies are needed to clarify this issue.

In conclusion, we used RNAi strategy to deplete MEF2C in the left ventricle of adult mice and understand the role of this factor in the responses of the left ventricle to mechanical stress. The data indicate that depletion of MEF2C is sufficient to markedly attenuate the hypertrophic growth and myocardial fibrosis of overloaded left ventricle by a mechanism dependent on defective activation of mTOR/S6K pathway. Overall, these data highlight the potential of MEF2C in the pathogenesis of cardiac hypertrophy and remodeling, and provide insights into novel therapeutic targets to heart disease.

## Materials and Methods

Expanded Materials and Methods are available in the Supporting Information Text S1 file of this manuscript.

### Ethics Statement

Animals were handled in compliance with the principles of laboratory animal care formulated by the Animal Care and Use Committee of University of Campinas. Procedures such as jugular vein catheterization, aortic banding, echocardiographic examination and arterial vessels catheterization for blood pressure monitoring were performed under anesthesia with a mixture of ketamine (100 mg/Kg) and xylazine (5 mg/Kg).

### Antibodies and Chemicals

Polyclonal mouse antibody against MEF2C was from Abcam (ab43796-100). Polyclonal rabbit antibodies against FAK (sc558), SHP2 (sc7384), JNK (sc571), GAPDH (sc25778), S6K p70 (sc230), pS6K p70 Thr389 (sc11759R) and PGC1-α (sc13067), were purchased from Santa Cruz Biotechnology (USA). Antibody against MEF2A was from Cell Signaling (9736). Antibodies were used following the manufacturer's recommendation. Colagenase type IA and trypsin were from Sigma (USA). Trizol, Phenol and Super Script II were from Invitrogen. Super Signal west Pico Cheluminescent Substract and Ampliscribe T7 high yield transcription were from Epicentre. L-Leucine was from Ajinomoto Aminoscience LLC.

### Experimental Models and Animals

Swiss mice (6–8 week old) and neonatal Wistar rats were obtained from the animal facility center of State University of Campinas. Animals were handled in compliance with the principles of laboratory animal care formulated by the university's Animal Care and Use Committee. Procedures such as jugular vein catheterization, aortic banding, echocardiographic examination and arterial vessels catheterization for blood pressure monitoring were performed under anesthesia with a mixture of ketamine (100 mg/Kg) and xylazine (5 mg/Kg) as previously reported[19]. Primary cultures of neonatal rat ventricular myocytes (NRVMs) were prepared from 1- to 2-day-old Wistar rats and plated on type I collagen Bioflex plates at 5×10^5^ cells/well, as previously reported[38].

### Isolation of Adult Mouse Ventricular Myocytes

Mouse cardiac myocytes were isolated from mice ventricle one day after treatment with siRNA targeted to MEF2C (siMEF2C) or GFP (siGFP), as previously reported[19].

### Echocardiography

2D M-mode echocardiography was performed with a 12-MHz probe connected to a Toshiba Power Vision system in anesthetized mice by a blinded observer at 15 minutes after the induction of anesthesia, as previously reported[19]. The short axis measurements were taken at the level of the midpapillary muscle. Three measurements were taken at end-systole and end-diastole to determine left ventricular diastolic and systolic diameter, wall thickness and fractional shortening.

### Hemodynamics

For blood pressure monitoring the right carotid and the right femoral arteries were cannulated with flame stretched PE-50 polyethylene tube. Blood pressure in the carotid and femoral arteries were simultaneously recorded for a 10 minute period to determine the transconstriction systolic gradient. The following parameters were computed: systemic systolic, mean and diastolic blood pressure (mmHg) and heart rate (bpm).

### Histological Examination

Hearts were rapidly excised from fully anesthetized mice and washed in PBS. The left ventricles were fixed in 10% paraformaldehyde, embedded in paraffin and cut into 5 µm sections. Tissue sections stained with haematoxylin and eosin (HE) and Masson's trichrome underwent morphometric studies using an image analysis system (Leica Q500 iW; Leica Imaging Systems, Cambridge, UK).

### siRNA Design and Synthesis

siRNA targeted to mouse MEF2C gene was designed and synthesized as previously published[18], [19]. DNA oligonucleotides (IDT-USA) were as follow: (i) T7: 5′-GGTAATACGACTCACTATAG-3′. (ii): MEF2C 1187 sense: 5′-CCCACCUGGCAGCAAGAACAC-3′ (iii): MEF2C 1187 antisense: 5′-GUUCUUGCUGCCAGGUGGGAU-3′ (iv): GFP sense: 5′-GTGTCTTGTAGTTCCCGTCTATAGTGAGTCGTATTACC-3′. (v): GFP antisense: 5′-ATGACGGGAACTACAAACACCTATAGTGAGTCGTATTACC-3′ were ordered from IDT (USA). The oligonucleotide-directed production of small RNA transcripts with T7 RNA polymerase were made with Ampliscribe™ T7 transcription kit (Epicentre Biotechnologies; Madison WI, USA) according to manufacturer's instructions.

### Transfection of NRVMs with siRNA

NRVMs were transfected with siRNA as previously reported [18].

### Western Blotting

Tissue or cell extracts containing equal amounts of total protein (50 µg) were resolved by 8% SDS-PAGE. The membranes were incubated with primary antibodies. Detection was accomplished by using an enhanced chemiluminescence detection system.

### Gene Transcripts and Mitochondrial DNA Quantification

Myocardial MEF2A, MEF2C, MEF2D and ANP transcripts were quantified after reverse transcription to cDNA followed by real-time PCR. Primers are shown in Table S1 of Supporting Information. Results were evaluated by the comparative CT method in respect to GAPDH, used as the normalizer. For mithocondrial and nuclear DNA quantification, were compared the expression of D-loop (EU194676.1) and 18S rRNA (NR_003278.1) genes, respectively.

### Myocardial AMP and ATP

Samples of myocardium were analyzed for AMP and ATP quantification by a method based on high performance liquid chromatography.

### Statistical Analysis

Data are presented as means±SEM. Student's *t*-test and 1-way repeated-measures ANOVA were used to compare groups. Post hoc analysis was performed with Bonferroni multiple-range test. A value of *P*<0.05 indicated statistical significance.

## Supporting Information

Text S1 (0.08 MB DOC)

Table S1 (0.04 MB DOC)
