# Correction: MEF2C Silencing Attenuates Load-Induced Left Ventricular Hypertrophy by Modulating mTOR/S6K Pathway in Mice

**DOI:** 10.1371/journal.pone.0112344

**Published:** 2014-10-27

**Authors:** The PLOS ONE Staff

Figure 3 is incorrect. The Western blot in Figure 3c should have four bands instead of five. The authors have provided a corrected version of Figure 3 here.

**Figure 3:**
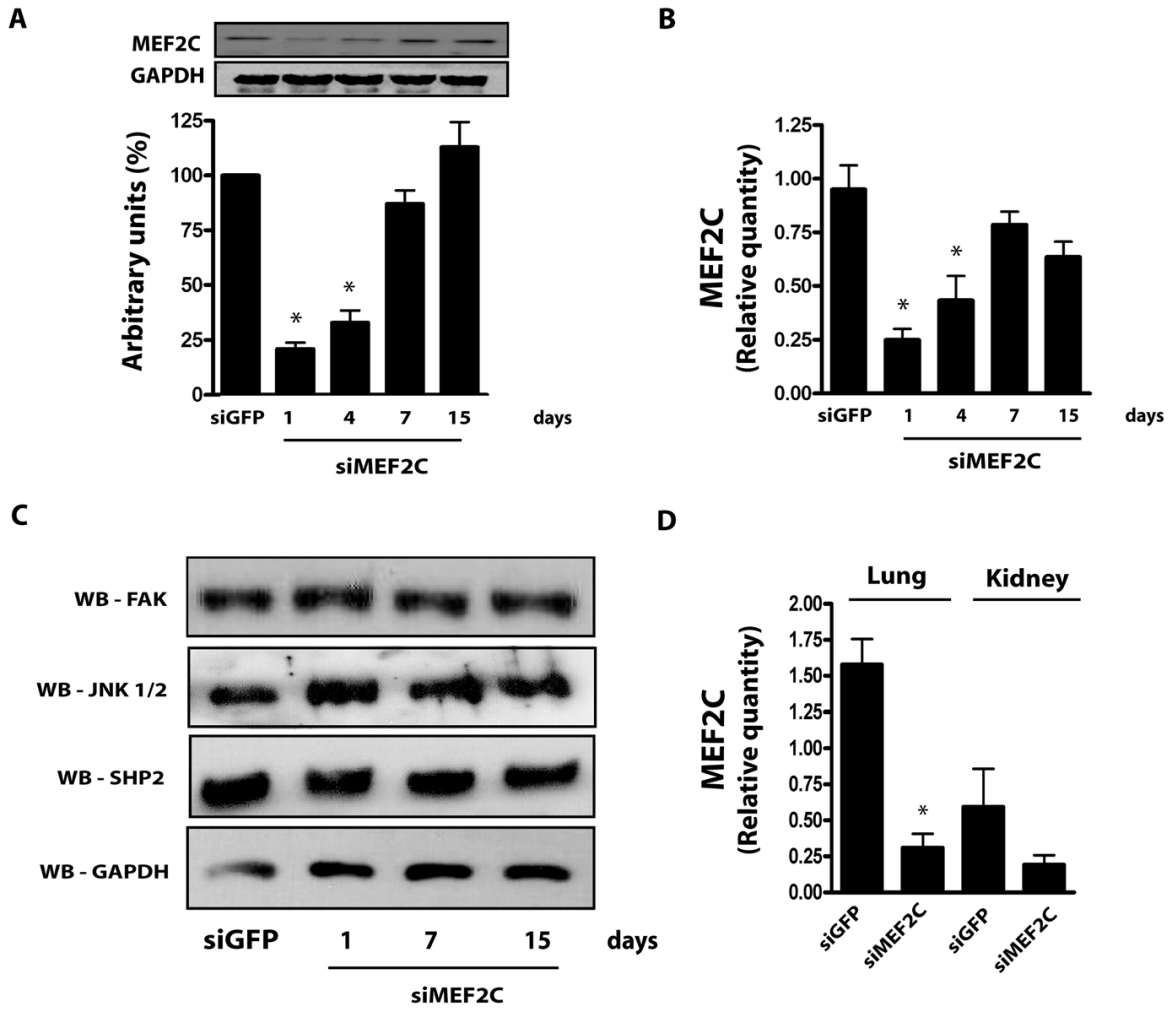
Effects of siMEF2C in distinct tissues and signaling proteins. (A) Time-course of MEF2C protein expression and (B) transcript levels normalized by GAPDH. (C) Myocardial expression of FAK, JNK, SHP2 and GAPDH. (D) Relative quantity of MEF2C transcripts in mice lung and kidney. * p<0.05 vs treatment with siGFP. At least 5 mice were employed for each subgroup.

## References

[1] Pereira AHM, Clemente CFMZ, Cardoso AC, Theizen TH, Rocco SA, et al (2009) MEF2C Silencing Attenuates Load-Induced Left Ventricular Hypertrophy by Modulating mTOR/S6K Pathway in Mice. PLoS ONE 4(12): e8472 doi:10.1371/journal.pone.0008472 20041152 10.1371/journal.pone.0008472 PMC2794538

