# Supplementary material for: MEF2C Silencing Attenuates Load-Induced Left Ventricular Hypertrophy by Modulating mTOR/S6K Pathway in Mice
Source: PLoS One. 2009 Dec 29;4(12):e8472. doi: 10.1371/journal.pone.0008472 (PMC2794538; doi:10.1371/journal.pone.0008472)
Supplement: Text S1 — (0.08 MB DOC) [file pone.0008472.s001.doc]

**SUPPORTING INFORMATION**

**Antibodies and Chemicals** Polyclonal mouse antibody against MEF2C was from Abcam (ab43796-100). Polyclonal rabbit antibodies against FAK (sc558), SHP2 (sc7384), JNK (sc571), GAPDH (sc25778), AMPK (sc25792), pAMPK Thr172-R (sc33524-R), S6K p70 (sc230), pS6K p70 Thr389 (sc11759R) and PGC1-α (sc13067), were purchased from Santa Cruz Biotechnology (USA). Antibody against MEF2A was from Cell Signaling (9736). Colagenase type IA and trypsin were from Sigma (USA). Trizol, Phenol and Super Script II were from Invitrogen. Super Signal west Pico Cheluminescent Substract and Ampliscribe T7 high yield transcription were from Epicentre.

**Experimental models and animals** Swiss mice (6–8 weeks old) and neonatal Wistar rats were obtained from the animal facility center of State University of Campinas (CEMIB). Animals were handled in compliance with the principles of laboratory animal care formulated by the university´s Animal Care and Use Committee. Procedures such as jugular vein catheterization, aortic banding, echocardiographic examination and arterial vessels catheterization for blood pressure monitoring were performed under anesthesia with a mixture of ketamine (100 mg/Kg) and xylazine (5 mg/Kg). For the injections of siRNA, the jugular vein was cannulated with a flame stretched PE50 polyethylene tube. Aortic banding was performed by transverse aortic constriction (TAC) by tying an 8.0 nylon (Dafilon; B.Braun Melsungen AG, Germany) suture ligature against a 27-gauge needle. Animals was treated with leucine 1,5% in drink water.

Primary cultures of neonatal rat ventricular myocytes (NRVMs) were prepared from 1- to 2-day-old Wistar rats, as previously reported^1^. Briefly, each culture used about 70 neonatal rat hearts. After the decapitation, the hearts were rapidly excised, rinsed in cold saline solution and minced coarsely. The myocytes were isolated by digestion with pancreatin and type 2 collagenase and then purified on a discontinuous Percoll gradient, suspended in plating medium containing 10% horse serum, 5% fetal bovine serum, and 0.5% penicillin-streptomycin, and plated in type I collagen Bioflex plates at 5 x 10^5^ cells/well. After 44 h, the medium was replaced with serum-free DMEM and incubated for 24 h under 95% air-5% CO2 before use.

**Isolation of adult mouse ventricular myocytes** Adult mouse cardiac myocytes were isolated from mice one day after treatment with siRNA^MEF2C^ or siRNA^GFP^, as previously published^2^. Mice were anesthetized and treated with 500 UI of heparin. The ascending aorta was cannulated for retrograde perfusion, right atrium was cut and the heart was perfused at 2.2mL/min with 5mL of perfusion buffer (118mM NaCl, 25mM NaHCO_3_, 1.2mM KH_2_PO_4_, 4.7mM KCl, 1.2mM MgSO_4_, 10mM Hepes free acid, 10mM glucose, 0.1mL/L insulin) and then switched to 30mL of perfusion buffer plus with 1g/L BSA and collagenase type IA (0.3 mg/mL; Sigma). The heart was quickly removed from the chest, minced in small pieces, and digested with 3mL of perfusion buffer with 1g/L BSA and trypsin (0.1mg/mL). Dissociated cells were centrifuged at 3.000 rpm for 5 minutes at the end of incubation and cell pellet was resuspended in DMEM (GIBCO) supplemented with 10% fetal bovine serum (GIBCO) and antibiotics (Penicillin and streptomycin, Nutricell). Cells were seeded into a 90mm plastic dish and incubated at 37°C in a humidified atmosphere of 5% CO_2_. After 2 hours, unattached cells that corresponded most to cardiomyocytes were harvested by centrifugation at 3000 rpm for 5 minutes. Cell pellet was then resuspended in buffer (100mM Tris-HCl pH 7.4; 100mM NaHPO_4_; 100mM sodium fluoride; 10mM EDTA; 10mM Na_3_VO_4_; 2mM PMSF; 0.2 mg/ml aprotinin, 10% Triton-X 100) and cleared by centrifugation at 11000 rpm, 4°C. Extracts containing 50μg total protein were resolved in SDS-PAGE and assayed by western blotting.

#### Echocardiography 2D M-mode echocardiography was performed with a 12-MHz probe connected to a Toshiba Power Vision system in anesthetized mice by a blinded observer at 15 minutes after the induction of anesthesia, as previously published^2^. The short axis measurements were taken at the level of the midpapillary muscle. Three measurements were taken at end-systole and end-diastole to determine left ventricular diastolic and systolic diameter, wall thickness and fractional shortening.

**Hemodynamics** For the blood pressure monitoring the right carotid and the right femoral arteries were cannulated with flame stretched PE-50 polyethylene tube. Blood pressure in the carotid and femoral arteries were simultaneously recorded for a 10 minute period to determine the transconstriction systolic gradient. The following parameters were computed: systolic, mean and diastolic blood pressure (mmHg) and heart rate (bpm). The recordings were obtained from a COBE transducer (Arvada, USA) and connected to a GP4A Stemtech amplifier (Stemtech, USA). The amplifier output was connected to an analog-to-digital board and this to a computer loaded with WINDAQ-PRO Data Acquisition software (DATAQ Instruments, USA), for continuous blood pressure monitoring. Pulsatile blood pressure from carotid and femoral catheters were recorded in individual channels and sampled at 100 Hz.

**Gravimetry** The left ventricle was removed, cleansed and weighed and the relative left ventricle weight index (mg/g) determined.

**Histological examination** Hearts were rapidly excised from fully anesthetized mice and washed in PBS. The left ventricles were fixed in 10% paraformaldehyde, embedded in paraffin and cut into 5 μm sections. Tissue sections stained with haematoxylin and eosin (HE) and Masson's trichrome underwent morphometric studies using an image analysis system (Leica Q500 iW; Leica Imaging Systems, Cambridge, UK). To estimate left ventricular hypertrophy, HE-stained sections were examined under x400 magnification. The myocyte diameter (width; in μm) was measured around oval and central nuclei of longitudinally displayed myocytes. To estimate left ventricular fibrosis, collagen volume fraction (%) was determined in Masson's trichrome -stained sections under x200 magnification. The collagen volume fraction was calculated as the percentage of blue-stained connective tissue areas per total myocardium, excluding perivascular areas.

**siRNA design and synthesis** siRNA targeted to mouse MEF2C gene was designed as previously published^2^. Briefly, the sequences were selected based on the highest trend to incorporate the antisense strand in the RISC complex. The sequence which was the most effective was then tested in mice. DNA oligonucleotides: (i) T7: 5’-GGTAAT ACGACTCACTATAG-3’. (ii): MEF2C 1187 sense: 5’-CCCACCUGGCAGCAAGAACAC-3’ (iii): MEF2C 1187 antisense: 5’-GUUCUUGCUGCCAGGUGGGAU-3’ (iv): GFP sense: 5’-GTGTCTTGTAGTTCCCGTCTATAGTGAGTCGTATTACC-3’. (v): GFP antisense: 5’-ATGACGGGAACTACAAACACCTATAGTGAGTCGTATTACC-3’ were ordered from IDT (USA). The oligonucleotide-directed production of small RNA transcripts with T7 RNA polymerase were made with Ampliscribe™ T7 transcription kit (Epicentre Biotechnologies; Madison WI, USA) according to manufacturer’s instructions.

**Transfection of NRVMs with siRNA** NRVMs were transfected with siRNA as previously published^3^. Briefly, NRVMs were cultured for 48 hours and then washed, starved from fetal serum and, after 24h, treated with siRNAs targeted to MEF2C or to GFP complexed with lipofectamine at a final concentration of 7nM, for 24h. After this period, cells were extracted and the extracts were assayed. siRNA-lipofectamine complexes were prepared according to manufacturer`s instructions as follows: 6μL of lipofectamine2000 reagent was added to 150μL OPTI-MEM and incubated for 45 min at room temperature. In a separate tube, 0.3μL siRNA (0.96μg/μL) was mixed with 20μL OPTI-MEM and incubated for 5min at room temperature. The content of the two tubes were incubated for 25min at room temperature to allow the formation of the transfection complexes (siRNA + lipofectamine). The total volume of the transfection complex (~ 200μL) was added to each well containing cells and medium (3ml DMEM).

**Western blotting** Heart was harvested in buffer (100mM Tris-HCl pH 7.4; 100mM NaHPO_4_; 100mM sodium fluoride; 10mM EDTA; 10mM Na_3_VO_4_; 2mM PMSF; 0,2 mg/ml aprotinin, 10% Triton-X 100) and cleared by centrifugation at 11000 rpm, 4°C. Extracts containing equal amounts of total protein (50μg) were resolved by 8% SDS-PAGE and transferred to nitrocellulose membranes. After blocking with 5% nonfat milk solution for 30 minutes, the membranes were incubates with primary antibodies in 3% nonfat milk solution overnight at 4°C. Detection was accomplished by using an enhanced chemiluminescence detection system and the membranes were exposed to XAR film. Equal protein loading was confirmed by Comassie staining of the gel.

**RNA and DNA extraction** For gene expression quantification, total RNA (2μg) was treated with DNase I (RNase-free, Roche Molecular Biochemicals), and reverse-transcribed with random hexamers using SuperScript II reverse transcriptase (Invitrogen) to generate cDNA. DNA extraction was performed by standard phenol/chloroform procedure DNA was according to previously published method^4^.

**Real time PCR** Two detection systems were used for quantitative real time (qRT)-PCRs, an Applied Biosystems 7500 sequence detection system by TaqMan method and MX3000P system (Stratagene, La Jolla, CA) using SYBR green chemistry (Invitrogen). The primers are presented in the table below. For TaqMan system the PCR conditions (400nM of each primer) were 50º C for 2 minutes; 95º C for 10 minutes; 95º C for 15 seconds, 60º C for 1 minute (40 cycles). For Sybrgreen, the condictions were 10 ng of reverse-transcribed RNA, each primer at 400 nM, and 6 μl of 2X SYBR Green PCR Master Mix (Invitrogen), and each sample was analyzed in triplicate. GAPDH was used as normalizer gene for transcripts quantification and for mithocondrial and nuclear DNA quantification was used 18S rRNA gene as the normalizer gene. Results were evaluated by the comparative efficiency-corrected comparative quantification method (MxPro QPCR software – Stratagene) (User Bulletin No. 2, PerkinElmer Life Sciences). Sequences and primers used in the real-time PCR assays are shown in Table S1.

**Myocardial AMP and ATP** Mice hearts were rapidly excised after anesthesis, washed with cold saline and ground to a powder under liquid nitrogen. The nitrogen of the vials was left to dry and the powdered tissues were quickly transferred to pre-cooled plastic tubes. Powdered samples were kept in the -80°C freezer until the analysis by HPLC (High performance liquid chromatrography). ATP and AMP were extracted from powdered tissues as follows: Samples of ~1.5 to 5.0 mg were homogenized in 1 mL of 50 mmol L^-1^ K_2_HPO_4_ and 25 mmol L^-1^citric acid (pH 4.5). The mixture was placed in a water bath (80ºC) during 2 minutes to deactivate any biological process that could degrade the ATP, ADP and AMP. After vortex-mixing, samples were added with additional 3 mL of 50 mmol L^-1^ K_2_HPO_4_ and 25 mmol L^-1^citric acid (pH 4.5) and then 1 mL of the suspension was centrifuged (8000 rpm, 4 minutes). Samples of 200 µL of this suspension were mixed with 20 µl of 2-chloroacetaldehyde solution and heated at 80°C for 20 minutes^5, 6^. Aliquots of 25 µl of the reaction mixture were then resolved by liquid chromatography. Chromatographic analyses were carried out on a Waters Alliance equipment series 2695 (Milford, MA, USA) equipped with quaternary pump, sampler manager, degasser, and Waters 2475 fluorescence detector model. The fluorescence of derivatized compounds (ATP, ADP, AMP and ADO) were detected with excitation and emission wavelengths set at 280 and 420 nm, respectively. Chromatographic separations of the compounds were achieved at room temperature, using a reversed-phase Cosmosil 5C18-MS column (150x4.6 mm i.d.; 5 µm particle size) with a Cosmosil guard column (5C18-MS 10x4.6 mm) purchased from Phenomenex (Torrance, CA, USA). The column was equilibrated and eluted under gradient conditions using a flow rate of 1.0 ml/min. The standards and samples were separated using a gradient mobile phase consisting of methanol (A) and a solution of 50 mmol L^-1^ KH_2_PO_4_ and 25 mmol L^-1^ citric acid (pH 4.5) (B), which was prepared immediately before use and filtered through a 0.45 µm filter (Millipore, Milford, MA, USA). The gradient condition was : 0–4 minutes 2% A; 4–12 minutes linear gradient 2–15% A; 17-18 minutes reconditioning step of the column was 2% A isocratic for 2 minutes. The chromatographic run time for each analysis was 20 minutes. Aliquots of 25 µl were injected into HPLC system. System control, data acquisition, and processing were performed with a PC-Pentium IV Processor personal computer from Dell, operated with Microsoft Windows XP version 2003 and Waters Empower 2002 chromatography software.

**REFERENCES**

**1.** Torsoni AS, Constancio SS, Nadruz W, Jr., Hanks SK, Franchini KG. Focal adhesion kinase is activated and mediates the early hypertrophic response to stretch in cardiac myocytes. *Circ Res.* 2003;93:140-147.

**2.** Clemente CF, Tornatore TF, Theizen TH, Deckmann AC, Pereira TC, Lopes-Cendes I, Souza JR, Franchini KG. Targeting focal adhesion kinase with small interfering RNA prevents and reverses load-induced cardiac hypertrophy in mice. *Circ Res.* 2007;101:1339-1348.

**3.** Marin TM, Clemente CF, Santos AM, Picardi PK, Pascoal VD, Lopes-Cendes I, Saad MJ, Franchini KG. Shp2 negatively regulates growth in cardiomyocytes by controlling focal adhesion kinase/Src and mTOR pathways. *Circ Res.* 2008;103:813-824.

**4.** Roberts I, Ng G, Foster N, Stanley M, Herdman MT, Pett MR, Teschendorff A, Coleman N. Critical evaluation of HPV16 gene copy number quantification by SYBR green PCR. *BMC Biotechnol.* 2008;8:57.

**5.** Katayama M, Matsuda Y, Shimokawa K, Tanabe S, Kaneko S, Hara I, Sato H. Simultaneous determination of six adenyl purines in human plasma by high-performance liquid chromatography with fluorescence derivatization. *J Chromatogr B Biomed Sci Appl.* 2001;760:159-163.

**6.** Kawamoto Y, Shinozuka K, Kunitomo M, Haginaka J. Determination of ATP and its metabolites released from rat caudal artery by isocratic ion-pair reversed-phase high-performance liquid chromatography. *Anal Biochem.* 1998;262:33-38.
