# Supplementary material for: MEF2C Silencing Attenuates Load-Induced Left Ventricular Hypertrophy by Modulating mTOR/S6K Pathway in Mice
Source: PLoS One. 2009 Dec 29;4(12):e8472. doi: 10.1371/journal.pone.0008472 (PMC2794538; doi:10.1371/journal.pone.0008472)
Supplement: Table S1 — (0.04 MB DOC) [file pone.0008472.s002.doc]

Table S1. Sequences and primers used in real-time PCR assays.

| **System** | **Gene** | **NCBI Reference Sequence** | **Specie** | **Sequence** |
| --- | --- | --- | --- | --- |
| **Taq Man** | MEF2C | NM_025282.2 | Mouse | Mm 00600423_M1 - Applied Biosystems |
|  | MEF2A | NM_001033713.1 | Mouse | Mm 00488969_M1 - Applied Biosystems |
|  | GAPDH | NM_008084.2 | Mouse | [Mm 03302249_G1](https://products.appliedbiosystems.com:443/ab/en/US/adirect/ab?cmd=ABAssayDetailDisplay&assayID=Mm03302249_g1&Fs=y&adv_phrase3=EXACT&adv_phrase2=EXACT&adv_phrase1=EXACT&assayType=GE&catID=601267&SearchRequest.Common.SortSpec=SPECIES+asc&searchValue=GAPDH&searchBy=all&adv_kw_filter3=ALL&srchType=keyword&adv_kw_filter2=ALL&adv_kw_filter1=ALL&inventoried=*&adv_query_text3=&searchType=keyword&adv_query_text2=&adv_query_text1=&adv_boolean3=AND&displayAdvSearchResults=null&SearchRequest.Common.ResultsPerPage=25&adv_boolean2=AND&adv_boolean1=AND&chkBatchQueryText=false&kwfilter=ALL&SearchRequest.Common.PageNumber=2&isSL=null&msgType=ABGEKeywordResults) - Applied Biosystems |
|  |  |  |  |  |
| **Syber Gren** | MEF2C | NC_005101.2 | Rat | (F) 5’- GCA AGC ACA CAA TGC CAT CA -3’  (R) 5’- ACT GGG GTA GCC AAT GAC TG-3’ |
|  | ANP | [NM_008725.2](http://www.ncbi.nlm.nih.gov/entrez/viewer.fcgi?val=NM_008725.2) | Mouse | (F) 5’-TCGTCTTGGCCTTTTGGCT-3  (R) 5’-TCCAGGTGGTCTAGCAGGTTCT-3’ |
|  | MEF2B | [NM_001017507.1](http://www.ncbi.nlm.nih.gov/nuccore/NM_001017507.1) | Rat | (F) 5’-GCTTCGCCTTCCTACCACCAG-3  (R) 5’-CACCGCGTCCCTCGTTGGTG-3’ |
|  | MEF2B | NM_008578.2 | mouse | (F) 5’-GCTTTGCCTTCTTACCATCAG-3  (R) 5’-TACAGCGTCCCTCGTTGGTG-3’ |
|  | MEF2D | NM_133665.3 | Rat | (F) 5’-CAGCAGCCACCTCAGCAACAG-3’  (R) 5’-GACAGTGAGAGCAGCACCCAC -3 |
|  | MEF2D | NM_133665.3 | mouse | (F) 5’-CAGCAGCCACCTCAGCAACAG-3’  (R) 5’-GACTGTGAGAGCAGCACCCAC -3’ |
|  | GAPDH | [NM_008084.2](http://www.ncbi.nlm.nih.gov/nucleotide/126012538) | mouse | (F) 5’-GGCATTGCTCTCAATGACAA-3’  (R) 5’-AGGGTGCAGGGAACTTTATT-3’ |
|  | GAPDH | [NM_017008.3](http://www.ncbi.nlm.nih.gov/nucleotide/110347607) | rat | (F) 5’- GGC ATT GCT CTC AAT GAC AA -3’  (R)5’- AGG GTG CAG GGA ACT TTA TT -3’ |
|  | d-Loop | EU194676.1 | Mouse | (F) 5’- GGTTCTTACTTCAGGGCCATCA -3’  (R) 5’-GATTAGACCCGTTACCATCGAGAT -3’ |
|  | 18S | [NR_003278.1](http://www.ncbi.nlm.nih.gov/entrez/viewer.fcgi?db=Nucleotide&dopt=GenBank&val=120444899) | Mouse | (F) 5’-TAGAGGGACAAGTGGCGTTCT -3’  (R) 5’-CGCTGAGCCAGTCAGTGT -3’ |
